# Endocytic and Recycling Endosomes Modulate Cell Shape Changes and Tissue Behaviour during Morphogenesis in *Drosophila*

**DOI:** 10.1371/journal.pone.0018729

**Published:** 2011-04-14

**Authors:** Ana Margarida Mateus, Nicole Gorfinkiel, Sabine Schamberg, Alfonso Martinez Arias

**Affiliations:** 1 Department of Genetics, University of Cambridge, Cambridge, United Kingdom; 2 Gulbenkian PhD Programme in Biomedicine, Oeiras, Portugal; University of Texas MD Anderson Cancer Center, United States of America

## Abstract

During development tissue deformations are essential for the generation of organs and to provide the final form of an organism. These deformations rely on the coordination of individual cell behaviours which have their origin in the modulation of subcellular activities. Here we explore the role endocytosis and recycling on tissue deformations that occur during dorsal closure of the *Drosophila* embryo. During this process the AS contracts and the epidermis elongates in a coordinated fashion, leading to the closure of a discontinuity in the dorsal epidermis of the *Drosophila* embryo. We used dominant negative forms of Rab5 and Rab11 to monitor the impact on tissue morphogenesis of altering endocytosis and recycling at the level of single cells. We found different requirements for endocytosis (Rab5) and recycling (Rab11) in dorsal closure, furthermore we found that the two processes are differentially used in the two tissues. Endocytosis is required in the AS to remove membrane during apical constriction, but is not essential in the epidermis. Recycling is required in the AS at early stages and in the epidermis for cell elongation, suggesting a role in membrane addition during these processes. We propose that the modulation of the balance between endocytosis and recycling can regulate cellular morphology and tissue deformations during morphogenesis.

## Introduction

Morphogenesis, a process that deals with the spatial arrangement of cells over time to generate the final shape of tissues, organs and thereby organisms, spans several scales of organization and relies on changes in the organization of groups of cells. There are different types of deformations (e.g folding, rolling or spreading) that contribute to different morphogenetic processes, but they all rely on changes at the single cell level, which result from the activity of basic cellular processes: cytoskeleton activity, cell adhesion and intracellular trafficking. These activities result from the behaviour and interaction of key molecules, such as Myosin, Actin, Microtubules, Cadherins, Integrins and Rab proteins. These molecules, which can be seen to configure functional modules [1], act as effectors in cellular morphogenesis. How the modulation of the cellular processes at the single cell level is translated into changes in the overall behaviour and shape of a tissue is one of the outstanding questions in morphogenesis. A second related question is how the modulation of a particular cellular activity produces different tissue deformations in several morphogenetic processes.

The process of Dorsal Closure (DC), the last step in the morphogenesis of the *Drosophila* embryo, is a suitable model system to study epithelial morphogenesis. DC bridges a discontinuity on the dorsal region of the epidermis that is initially filled by a second epithelium, the amnioserosa (AS), that does not contribute to the larva and eventually undergoes apoptosis [2], [3]. The process of DC is accompanied by a reduction of the surface of the AS and the bilateral extension of the epidermis towards the dorsal midline, where closure will occur by dislodgement of the AS and fusion of the two epidermal sides [4]. The main forces that drive this process are generated by the contraction of the AS and the activity of an actin cable that forms at the Leading Edge (LE) of the epidermis [5], [6]. In contrast, the epidermis exerts a resistive force that opposes closure [5], [6]. The cellular dynamics of both AS and epidermal cells and the tissue deformations that they experience during DC have to be integrated to give rise to the stereotyped morphogenetic movement. Therefore, this process provides a good system to study how cellular mechanisms operate at the single cell level in different epithelia to generate different tissue behaviour.

The role of the cytoskeleton and cell adhesion systems during morphogenesis has been extensively explored [7], [8], . However, less is known about the role of the trafficking machinery in tissue movements and organ shape because intracellular trafficking has been regarded as a passive process in this context [11]. In the last years some studies have addressed this issue [12], [13], [14], [15], [16] and are starting to reveal the requirements for the trafficking machinery during morphogenesis. The intracellular trafficking can be coarsely divided into the biosynthetic pathway and the endocytic pathway [17]. The biosynthetic pathway has been shown to play an important role in morphogenesis [11], [12], but here we focused on the endocytic pathway, which removes and recycles back proteins and membrane from the cell surface. The endocytic pathway has been implicated in the remodelling of adhesive molecules at the plasma membrane [13], [15], [18], [19], [20], in the generation of membrane flows that allow membrane growth [12], [21], [22], [23], [24], [25], [26] and in the organization of membrane domains that can then modulate cytoskeletal organization [27], [28], [29], [30], [31]. The above studies suggest that the endocytic pathway could also play a role in the changes in cell and tissue behaviour observed during DC.

The endocytic pathway is organized into three major compartments, each characterized by a specific Rab protein [32]: early endosomes, receive products of endocytosis and are enriched in Rab5; late endosomes, define a route for degradation and are associated to Rab7; and recycling endosomes, which allow the return of recently endocytosed proteins to the membrane, are associated with Rab11 [33], [34]. Rab11 endosomes are also used by the biosynthetic pathway to insert new proteins into the cell membrane [18], [35]. Due to their selective enrichment in particular compartments, Rab proteins can be used as tags for the different steps of the endocytic pathway [32] and interfering with their function through mutations or Dominant Negative (DN) forms, enables blocking the endocytic pathway at different steps. Mutants often have widespread effects and therefore the use of DN can be helpful in unravelling specific functions of these proteins. DN constructs of GTPases can be unspecific and impair the function of other proteins [36], [37], [38], [39] and therefore should be used judiciously. However, the effects of Rab5^DN^ and Rab11^DN^ have been shown to reflect RNAi mediated loss of function, which suggests that the DN proteins specifically blocks Rab function [40], [41], [42].

Here we have explored the role of endocytosis and recycling in the AS and the epidermis during DC. Our results show for the first time that there are different requirements for endocytosis and recycling in the epidermis and AS and that each activity is associated with different tissue deformations. These observations led us to propose a model in which the balance between endocytic and recycling activities can modulate cellular morphology by controlling membrane flow during morphogenesis.

## Results and Discussion

### Rab5 and Rab11 exhibit planar polarization in the epidermis

**Figure 1:**
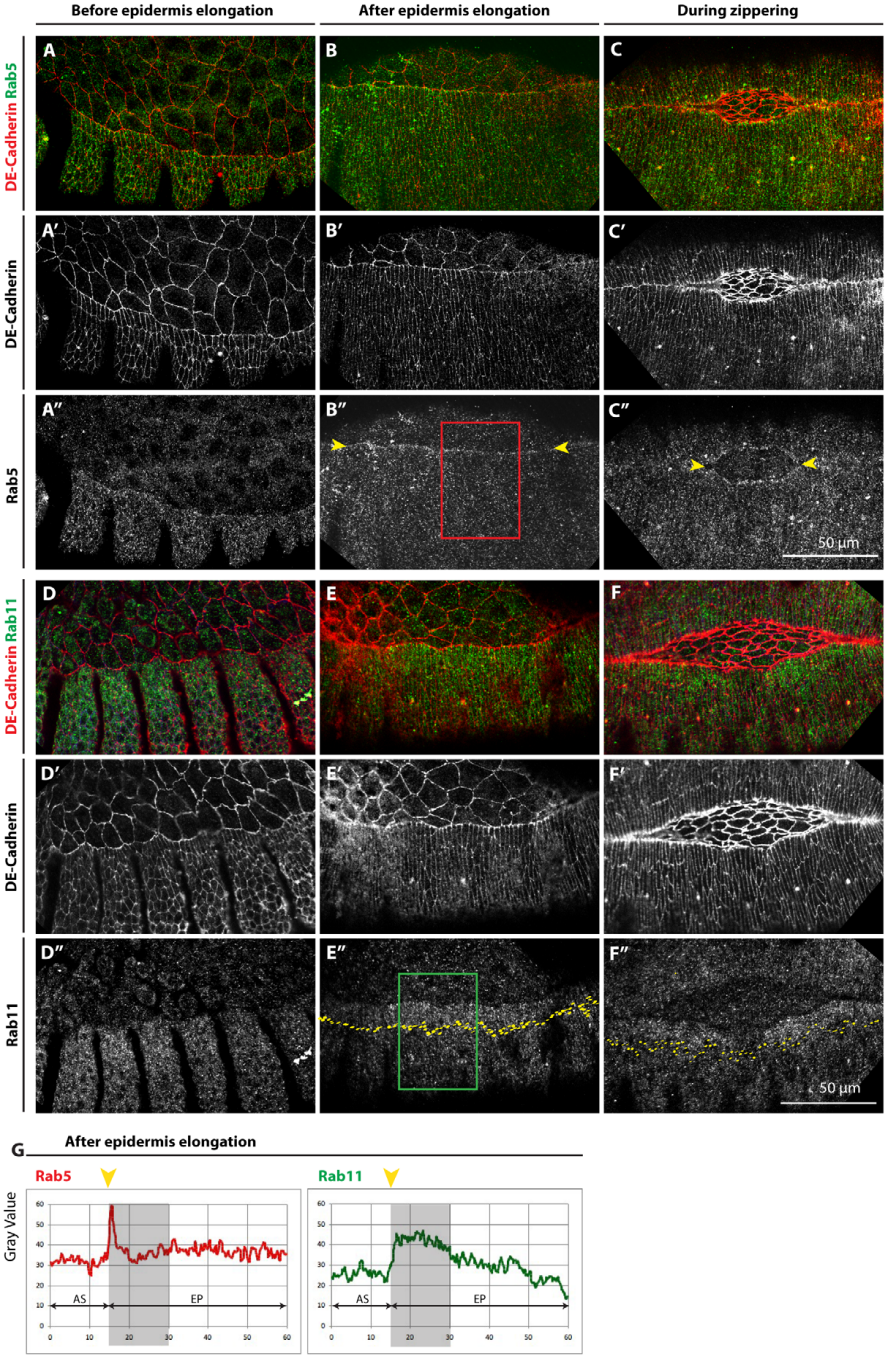
Rab5 and Rab11 are planar polarized in the epidermis. (A–F″) Confocal sections from wild type *Drosophila* embryos immunostained with DE-Cadherin, Rab5 (A–C) and Rab11 (D–F) antibodies, before epidermal cell elongation (first column of panels), after epidermal cell elongation (second column of panels) and during zippering (third column of panels). Rab5 vesicles accumulate at the LE, especially after epidermal cell elongation (B″, arrowheads), and this is maintained during zippering (C″, arrowheads). There is an enrichment of Rab11 labelled vesicles in the DME cells after epidermal cell elongation (E″) that is also maintained during zippering (F″, yellow dots indicate the borders of DME cells). Quantification of fluorescence levels from boxes B'', E'' (G). Vesicles labelled for Rab11 are more abundant in epidermal cells than in the AS cells, but not Rab5 (G). Yellow arrow indicates the LE, grey box the approximated location of the DME cells.

To study the role of the endocytic pathway in DC we first analyzed the organization of different endosomes in the epidermis and the AS of the *Drosophila* embryo by following the distribution of Rab proteins associated with early (Rab5), recycling (Rab11) and degradation (Rab7) endosomes [32] with specific antibodies. Before the onset of DC, Rab5 is in dots randomly distributed within epidermal and AS cells (Figure 1A″). However, as epidermal cells begin to elongate, Rab5 positive vesicles accumulate at the LE of the Dorsal Most Epidermal (DME) cells and this distribution is maintained throughout the rest of the process (arrowheads, Figure 1B″, 1C″). Subcellular asymmetries were not observed in the distribution of the recycling endosomes, although the levels of Rab11 labelled endosomes appeared higher in the DME cells after epidermal cell elongation and during the zippering of the two opposing epithelia (Figure 1D″,E″,F″). Quantification of Rab5 and Rab11 confirms these observations and also shows that the levels of Rab5 are the same in the AS and the epidermis, except at the LE, whereas Rab11 is expressed at higher levels in the epidermis (Figure 1G). In contrast, Rab7 exhibited a homogeneous distribution throughout DC (not shown).

Altogether, these observations indicate that the organization of endocytic and recycling endosomes in the epidermis is spatially and temporally regulated during DC. To assess whether the activity of these proteins is implicated in the changes in cell behaviour underlying this morphogenetic event, we expressed DN forms of Rab5 and Rab11 separately in the AS and the epidermis to block endocytosis (Rab5) and recycling (Rab11). We did not analyse Rab5 or Rab11 loss of function mutant alleles due the perdurance of the maternal contribution until DC stage, which does not allow a complete assessment of Rab5 and Rab11 function (unpublished observations and [44]). Moreover, we were interested in gauging the contribution of trafficking in each participating tissue, which is only possible by tissue specific expression of the Rab DN forms using the UAS/GAL4 system (see below and Figure S1). We focused our study on cell shape changes, and DE-Cadherin and Actin cytoskeleton organization, which play central roles in the process [45], [46], [47], [48], [49], [50], [51], [52], [53]. Since DE-Cadherin is enriched at Adherens Junctions (AJ) in the apical region of cells, it was also used to follow apical cell shape and tissue dynamics.

### Blocking Rab5 function changes AS cell morphology, but does not impair epidermal cell elongation and fusion

**Figure 2:**
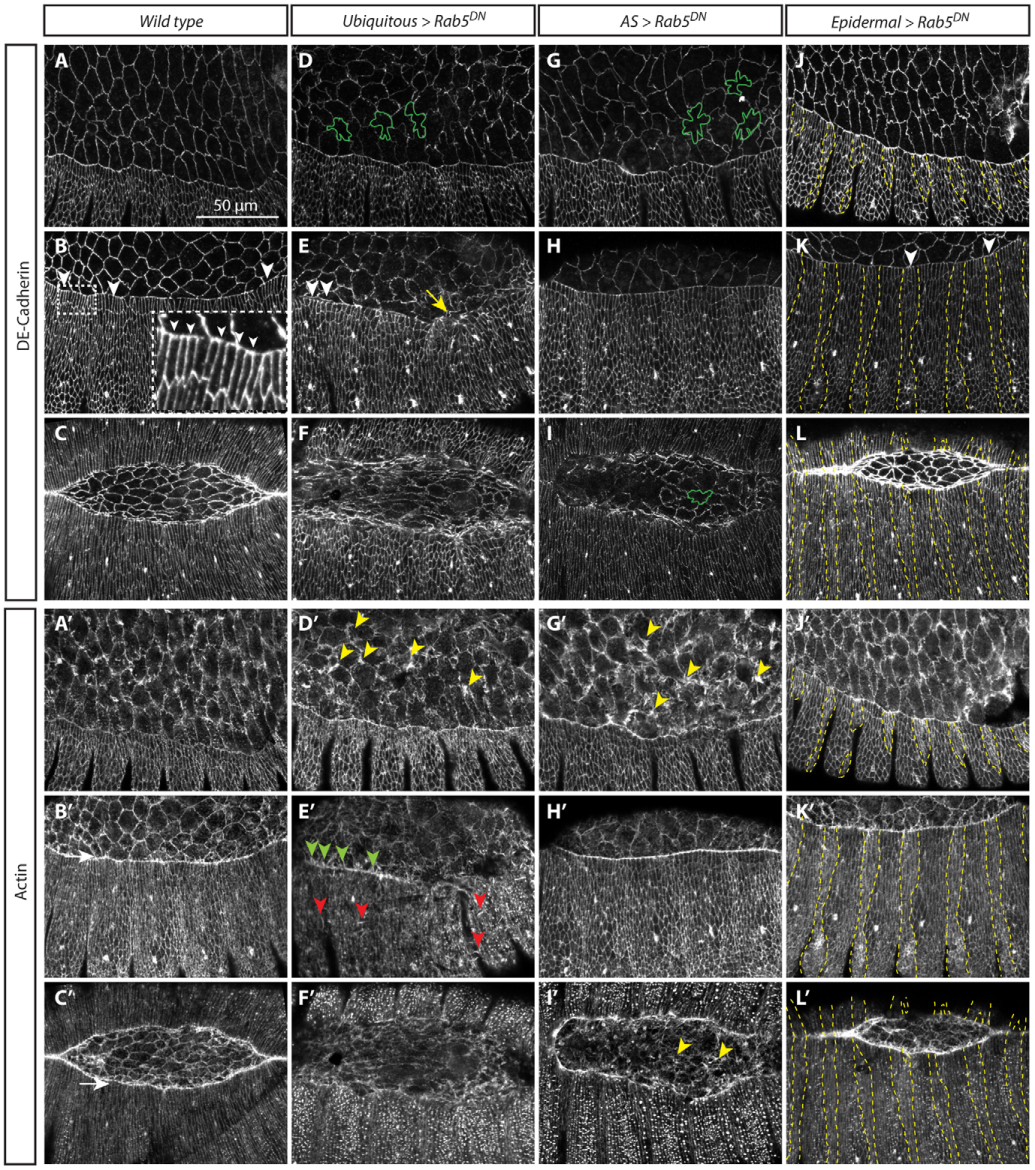
Blocking endocytosis affects actin organization, but not DE-Cadherin levels. Confocal sections from *Drosophila* embryos immunostained with DE-Cadherin (A–L) and phalloidin to visualize F-actin (A'–L'). (A–C'). Wild type embryos during different DC stages show the dynamic distribution of DE-Cadherin and F-actin. Arrowheads in inset B highlight the clearance of DE-Cadherin from the LE and accumulation at the ANCs. In this and all subsequent genotypes three different stages of DC are shown (D–F'). Early stages of DC embryos expressing Rab5^DN^ ubiquitously show irregular AS cell shapes (D, green contours) and big lamellipodia (D', yellow arrowhead). Later during DC, these embryos exhibit impaired epidermal elongation and puckering (E, yellow arrow) and a strong actin cable with bigger and more filopodia (E', green arrowheads). Actin projections are also observed throughout the epidermis (E', red arrowheads). Zippering is impaired in these embryos (F, F'). (G–I') Embryos expressing Rab5^DN^ in the AS results in changes in cell morphology, particularly evident at early and late stages of DC (G,I), but epidermal cell elongation is not compromised (H). Zippering is also affected (I). Abnormal filopodia and lamellipodia are observed in the AS (G', I', yellow arrowheads) throughout DC. Embryos expressing Rab5^DN^ in the engrailed domain do not exhibit defects in epidermal cell elongation nor in zippering. DE-Cadherin shows a wild type distribution (J–L), but stronger levels of Actin are observed in the engrailed domain (J', K'). Rab5^DN^ domain of expression is enclosed in the yellow dashed line (J–L').

In wild type embryos, the onset of DC is characterized by a scalloped interface between the LE and the AS (Figure 2A). At this stage, DE-Cadherin and Actin are homogeneously distributed at the membrane and cell cortex of both epidermal and AS cells (Figure 2A). As DC starts, the AS begins to reduce its area and the epidermis initiates its elongation in the Dorsal/Ventral (D/V) direction. At this time, the expression of DE-Cadherin is reduced at the LE (Figure 2B), where Actin filaments assemble a supracellular cable (Figure 2B,C). A particular feature of this phase of DC is the progressive emergence of filopodia and lammellipodia from the Actin rich LE. During the later stages of DC, the opposing epithelia meet at the dorsal midline, the filopodia interdigitate and zippering occurs leading to epithelial continuity (Figure 2C).

#### Cell structure and organization

To assess the role of the endocytic machinery in DC, we first blocked endocytosis by expressing a Rab5 dominant negative molecule (Rab5^DN^) ubiquitously during embryogenesis. In this situation, the morphology of the AS cells is affected, epidermal elongation is impaired (Figure 2D–F’) and DC fails: the embryos exhibit a conspicuous dorsal hole in the cuticle (Figure S2B). A most clear feature of these embryos is an apparent expansion of the cell surface of the cells of the AS which appears corrugated. Similar defects in AS cell morphology are observed when Rab5^DN^ is expressed only in the AS (Figure 2G) but in this instance the epidermal cells elongate and the embryos complete DC (Figure S2A). Expression of Rab5^DN^ only in the epidermis, with the enGAL4 driver, has no visible effects on DC or cell activity (Figure 2J–L).

#### Adhesion and cytoskeleton

Although an increase in DE-Cadherin levels has been observed when Rab5 function is impaired [14], [15], [55], [56], we did not observe this either in the epidermis or in the AS upon expression of Rab5^DN^. Furthermore, in these embryos DE-Cadherin is cleared from the LE as in wild type embryos (Figure 2B,E,K and see also [16]). This suggests context dependent relationships between trafficking and DE-Cadherin. In contrast, cells expressing Rab5^DN^ exhibited an abnormal Actin organization. This effect is particularly evident in the AS, where cells exhibit exuberant lamellipodia (Figure 2A’,D’). If expressed ubiquitously in the embryo, Actin projections can also be seen throughout the epidermis (Figure 2E’) and higher levels of Actin are detected when Rab5^DN^ is expressed in stripes in the epidermis (Figure 2J’,K’). Actin accumulation upon downregulation of Rab5 activity has also been observed in *Drosophila* oocytes: in *Rab5^2^* germ line clones, ectopic actin fibers were observed around early endocytic vesicles [57]. In contrast there is also evidence for the requirement of Rab5 activity in promoting lamellipodia formation and circular ruffling [58], [59]. These observations suggest that the effects of Rab5 on actin organization are context dependent. The effects on Actin organization during DC are specific to Actin, as we did not observe any changes in the distribution and organization of the Microtubule network or Myosin II (not shown).

#### Cell and tissue dynamics

**Figure 3:**
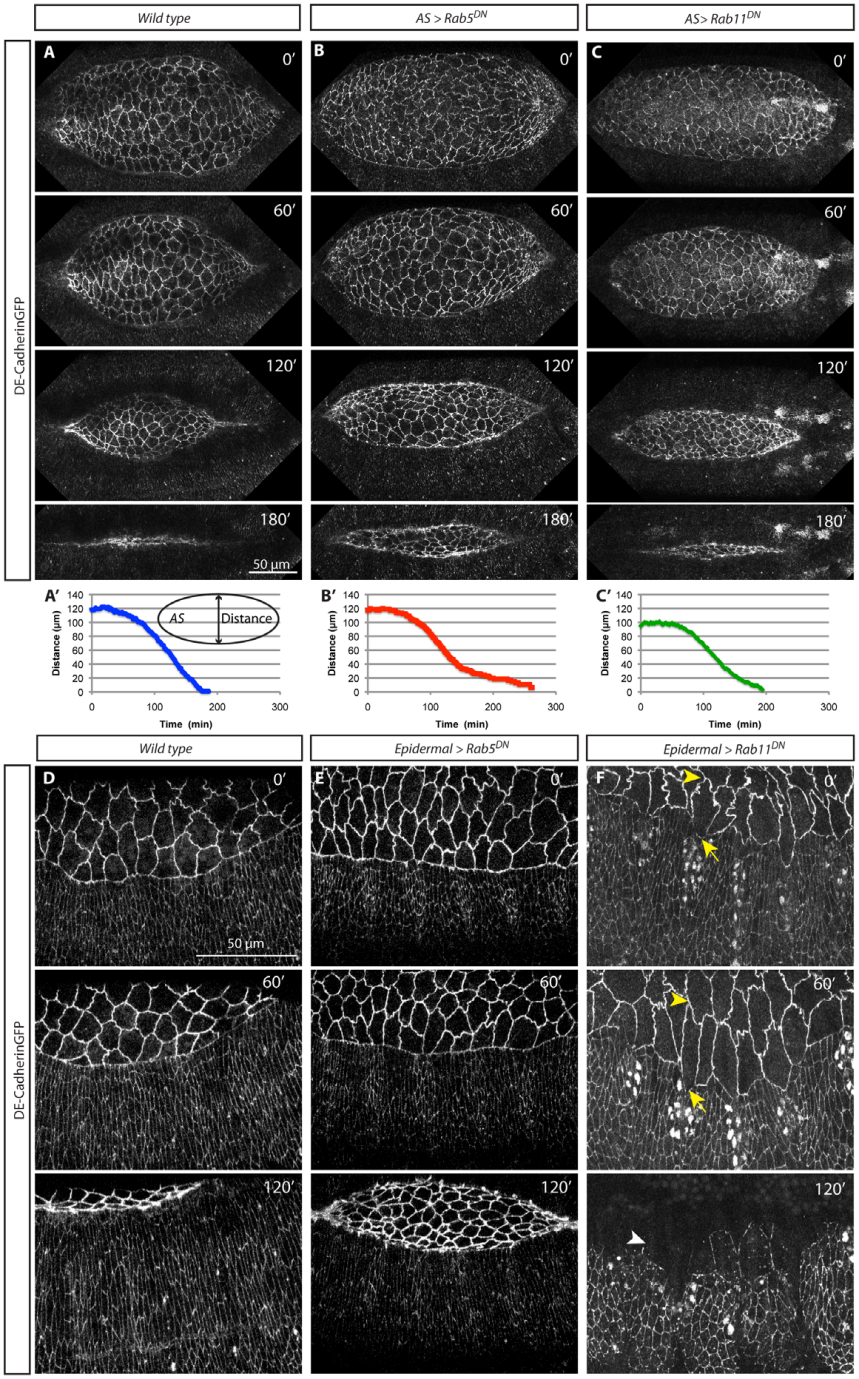
AS and Epidermal dynamics upon expression of Rab5^DN^ or Rab11^DN^. Still images from a time-lapse movie of a wild type embryo (A and Movie S1, D and Movie S4), an AS>Rab5^DN^ (B and Movie S2), an AS>Rab11^DN^ (C and Movie S6), an Epidermal>Rab5^DN^ (E and Movie S5), and an Epidermal>Rab11^DN^ (F and Movie S7) embryos, expressing ubi-DE-CadherinGFP, at different stages after the start of DC (indicated by the time stamp). Notice the irregular shapes of AS cells in B and C. No defects are observed in Epidermal>Rab5^DN^ embryos (E). Epidermal>Rab11^DN^ embryos exhibit a delay in the advancement of epidermal cells (F-0' arrows, HistoneYFP shows Rab11^DN^ domain of expression) and AS cells are very elongated in the D/V direction, in particular the ones facing the engrailed domain (F-60' yellow arrowheads). Ultimately the epidermis detaches from the AS (F-120', arrowhead). (A'–C') Measurement of the distance between the two opposing epithelia for a wild type (A'), an AS>Rab5^DN^ (B') and an AS>Rab11^DN^ (C') embryo. Closure is slower in AS>Rab5^DN^ embryos than in wild type ones (B'). Epidermal>Rab11^DN^ embryos exhibit a narrower AS (C') due to cell shape defects (see Fig. 5).

While the analysis of fixed tissue provides insights into the structure of the cells and its molecular underpinning, it does not reveal the dynamics of cellular ensembles, which is central to the process of DC. To monitor the dynamics of the system we performed live imaging of embryos ectopically expressing Rab5^DN^ using ubi-DE-CadherinGFP as an apical membrane marker (Figure 3A,B).

In wild type embryos AS cells are initially elongated in the dorso-ventral (D/V) direction and they progressively reduce their apical surface area by contracting mostly in this orientation [60]. During the process, cell interfaces in the AS acquire a wavy appearance that evolves towards a straighter shape, probably due to an increase in cortical tension [61]. Finally, during zippering AS cells contract both in the D/V and antero-posterior (A/P) directions, thus developing a more isometric shape resulting in an efficient contraction of AS tissue and closure of the hole ([60], [61]; Figure 3A and Movie S1). When Rab5^DN^ is expressed in the AS, cells display highly wavy apical membranes that correlate with the corrugations observed in fixed tissue (Figure 3B–0’ and Movie S2). During the zippering stage, we observe unusually large cells that seem to have had their apical constriction affected (Figure 3B-120’). In some embryos a few cells exhibit wiggly membranes at a point in which they should have already acquired straight membranes (Movie S3), supporting the idea that these cells have defects in removing membrane during apical constriction. Despite this, cells manage to constrict apically, but with a delay compared to wild type embryos. The kinetics of the process is affected (Figure 3B’) and the zippering is defective. We measured the rate constant of zippering using the rate-process model of DC [6] and have found that the zippering rate (K_z_) for embryos expressing Rab5^DN^ in the AS (K_z_ = 8.16 and 8.77 nm/sec) is lower than in wild type embryos (K_z_ = 15.62 and 13.64 nm/sec).

These results suggest that the apical contraction of AS cells is tightly linked to the removal of the apical membrane through Rab5-mediated endocytosis, even though AS cells constrict upon Rab5^DN^ expression. Assays with Dextran show that Rab5^DN^ construct blocks endocytosis in the epidermis of *Drosophila* embryos (Figure S3A–C), but we cannot discard the possibility that there is still some Rab5 activity. Moreover, endocytosis independent of Rab5 [62], [63] or other mechanisms might be implicated in AS apical constriction. We observed fine structures projecting from AS cell boundaries that localize just basal to the AJs (Figure S3D,E). These structures labelled with DE-CadherinGFP could be filopodia or membrane invaginations used to progressively remove bulk membrane during apical constriction. These DE-Cadherin labelled projections were detected in both wild type and AS> Rab5^DN^ embryos, suggesting that they are Rab5 independent. We also observed apical membrane folds that progressively fuse to form thick projections, which could be another mechanism to rapidly remove membrane from the apical surface area (Figure S3F–I). Altogether, our results show that one of the mechanisms for cell membrane removal is Rab5-mediated endocytosis but also suggests that other Rab5-independent processes could be acting.

A recent study has also shown a requirement of Rab5 or Dynamin activity for apical cell constriction in *Xenopus* gastrulation [64]. However, in DC, expression of Dynamin^DN^ results in cellular phenotypes that resemble Rab11^DN^, and not Rab5^DN^ (see below, Figure S4), confirming previous observations showing that Dynamin is also required for recycling [12].

In contrast with the observations in the AS, ectopic expression of Rab5^DN^ in epidermal stripes using the enGal4 driver, does not impair epidermal cell elongation or DC (Figure 3E). In wild type embryos the epidermis elongates, the LE becomes taut and there is a uniform advancement of the LE (Figure 3D and Movie S4). A similar epidermal progression is observed in Epidermal>Rab5^DN^ embryos (Figure 3E and Movie S5). Only occasionally, we observed small kinks in the epidermal stripes at the LE where Rab5^DN^ is expressed, but as zippering starts the LE becomes very uniform.

Non-homogeneous expression of Rab5^DN^ in the epidermis or different expression levels of the Gal4 drivers used could explain the impaired epidermal elongation when Rab5^DN^ was expressed ubiquitously but not when restricted to the epidermis. However, the importance of interactions between the AS and the epidermis has been shown by experiments where genetically and physically manipulated embryos have revealed DC to fail only when both tissues were affected, but if only one tissue is affected DC completes [6], [65], [66]. Therefore, it might be possible that when both tissues express Rab5^DN^ epidermal cell elongation is compromised, but if there is wild type Rab5 activity in the epidermis this tissue elongates and DC proceeds. This suggests a low requirement of Rab5 activity in the epidermis, only revealed when Rab5^DN^ is expressed in both tissues.

In summary, blocking Rab5 function does not affect DE-Cadherin levels but affects Actin organization. In addition, Rab5 activity is strongly required in the AS and it appears to be necessary for membrane removal during apical AS cell constriction. However, a low requirement of Rab5 activity for epidermal elongation is revealed when both tissues are affected. In light of these results it becomes important to assess the outcome of blocking membrane insertion at the plasma membrane. To explore this, in the next section we addressed the role of recycling endosomes in the cell shapes changes that underlie epidermal cell elongation and AS cell contraction.

### Blocking Rab11 mediated recycling affects AS cell shape and impairs epidermal cell elongation

To study Rab11 function during DC, we expressed a dominant negative form of Rab11 (Rab11^DN^) that blocks recycling.

#### Cell structure and organization

**Figure 4:**
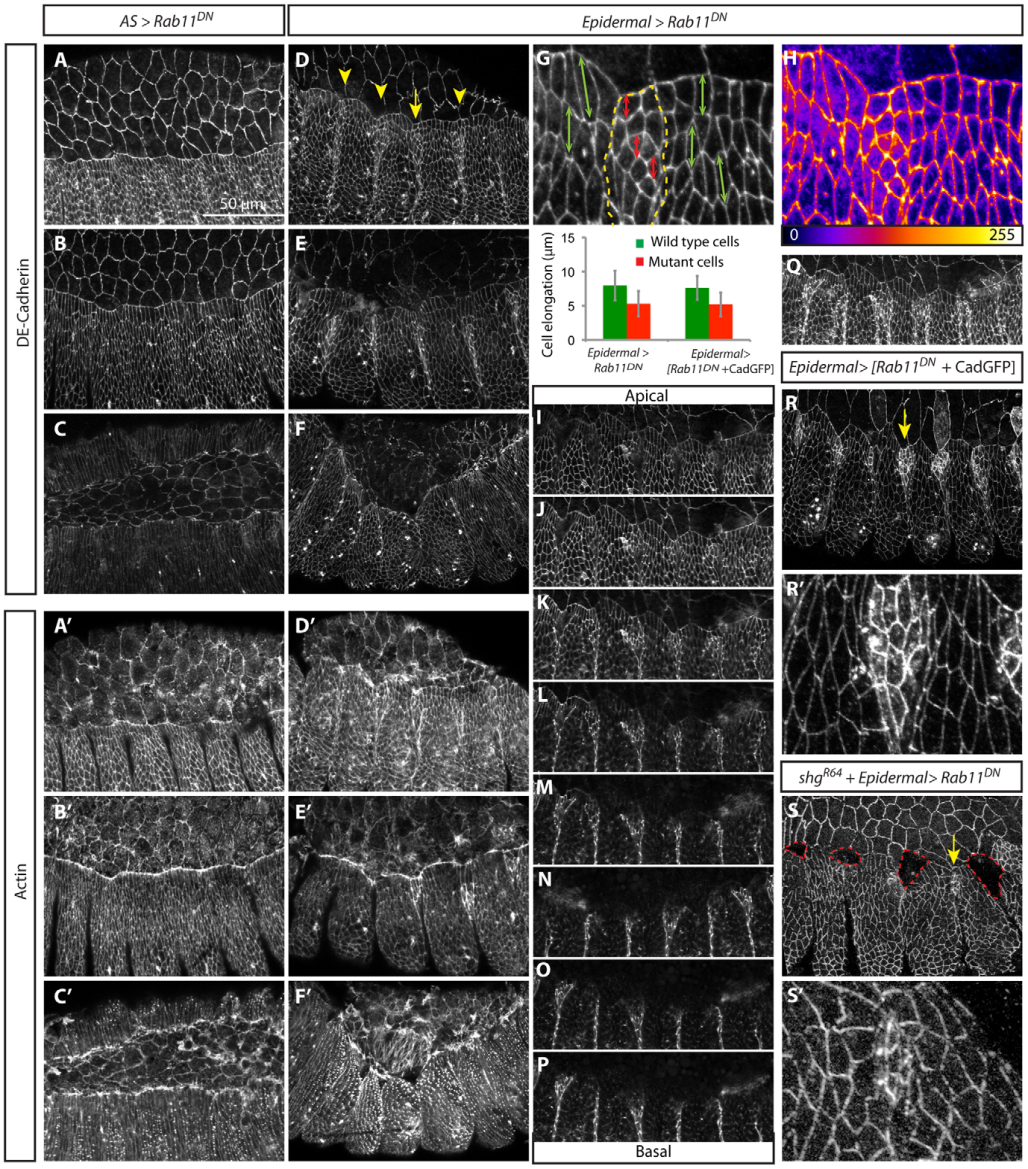
Rab11^DN^ expression impairs epidermal advancement and mislocalizes DE-Cadherin. Confocal sections from *Drosophila* embryos expressing Rab11^DN^ immunostained with DE-Cadherin (A–Q, R–S') and phalloidin to visualize F-actin (A'–F'). Three different stages of DC are shown (A–F'). At early stages, AS cells expressing Rab11^DN^ do not exhibit wavy membranes as in the wild type (A). Expression of Rab11^DN^ does not impair epidermal cell elongation (B), but zippering is deffective (C). DE-Cadherin levels (A–C) and Actin distribution (A'–C') in the AS are not affected. Embryos expressing Rab11^DN^ in the engrailed domain show a delay in the advancement of the dorsal epidermal cells and are less elongated (D, arrowheads). Later embryos exhibit ectopic grooves in the Rab11^DN^ domain of expression (E) and zippering does not occur (F). Actin accumulates at the LE in the DME cells expressing Rab11^DN^ (E'). Epidermal cell elongation in Rab11^DN^ expressing cells and wild type cells (H-yellow dashed line outline Rab11^DN^ domain of expression) and quantification of cell elongation of DME cells in the D/V direction in wild type cells (green) and Rab11^DN^ expressing cells (red) in Epidermal > Rab11^DN^ embryos (G - Magnification from D). Cell elongation is significantly reduced in Rab11^DN^ expressing cells [p<0.01 (N = 234 and 145 (G)]. In the mutant cells the levels of DE-Cadherin are higher at the cell membrane and in the cytoplasm of epidermal cells (H - Magnification from D, gradient represents fluorescence intensity values). DE-Cadherin is dispersed along the A/P axis in epidermal cells where Rab11^DN^ is expressed (I-P). Projection of sections I-P (Q). Overexpression and reduction of DE-Cadherin in embryos expressing Rab11^DN^ in epidermal stripes (R-S'). Epidermal cells expressing Rab11^DN^ and CadherinGFP exhibit a delay in epidermal advancement and impaired cell elongation similar to embryos expressing Rab11^DN^ alone in epidermal stripes [R, R', G (N = 129 and 69)]. In *shg^R64^* mutants (reduction of DE-Cadherin levels) epidermal cells expressing Rab11^DN^ do not elongate and detach from the AS (S, red dashed line outlines the holes). The levels of DE-Cadherin at the cell membrane are higher in the region where Rab11^DN^ is expressed (R', S').

Embryos expressing Rab11^DN^ ubiquitously display a loss of epidermal integrity reflected in the crumbled cuticle laid by these embryos, which has been described before (Figure S2D, see also [16]). This phenotype has been interpreted as a requirement of Rab11 in maintaining Crumbs and Adherens Junctions (AJs), ensuring epithelial integrity [16]. However, when Rab11 function was blocked only in the AS, these cells contracted and DC was completed (Figure 4A–C, Figure S2E). In contrast, expression of Rab11^DN^ in epidermal stripes impaired epidermal elongation (Figure 4G) and led to a delay in the advancement of the LE towards the dorsal midline, which eventually stalled (Figure 4D–F). This can be clearly seen in the mosaics generated by the expression of Rab11^DN^ in stripes using enGAL4. In these embryos we observed the formation of deep ectopic segmental grooves (Figure 4E,F) also evident in the cuticles, which in addition exhibit a large dorsal hole (Figure S2F).

#### Adhesion and cytoskeleton

Expression of Rab11^DN^ in the epidermis has no visible effect on Actin, it accumulates at the LE of the DME cells as in wild type embryos (Figure 4E’), suggesting that the non-uniform advancement of the epidermis cannot be attributed to defects in the actin cable [50]. These embryos exhibit an accumulation of DE-Cadherin in the cytoplasm of epidermal cells reflecting the lack of protein recycling. In addition, the levels of DE-Cadherin are also clearly increased at the cell membrane (Figure 4H). A closer inspection reveals that DE-Cadherin localization spans a larger domain along the apico-basal axis of epidermal cells (Figure 4I–P). This observation is in agreement with the suggestion that DE-Cadherin is first inserted at the basolateral membrane from where it is subsequently trafficked to the AJs through the activity of Rab11 [67].

Since modulation of the distribution of DE-Cadherin has been implicated in cell shape changes [13], [15] we tested whether the observed defects in DE-Cadherin localization could be the root of the lack of epidermal cell elongation. We reasoned that if DE-Cadherin recycling is required to promote epidermal cell elongation, adding or removing a copy of DE-Cadherin in embryos impaired in recycling would have an effect on the phenotype. Expression of Rab11^DN^ concomitantly with UASDE-CadherinGFP or in ubi-DE-CadherinGFP did not significantly change the cell elongation phenotype (Figure 4R,R’,G,3F). This phenotype persists when we expressed Rab11^DN^ in *shg^R64^* mutant embryos (*shg^R64^* is a null allele of DE-Cadherin [68]; in *shg^R64^* zygotic mutants DME cells elongate and initiate zippering, but the process is defective; cuticle preparations of the embryos exhibit small holes and mismatches [47], Figure S2K). However, when Rab11^DN^ is expressed in the engrailed domain of these mutant embryos there are detachments at early stages of DC, in the region where the DN is expressed (Figure 4S,S’). The cuticles from *shg^R64^* mutant embryos expressing Rab11^DN^ in the engrailed domain also exhibit a stronger phenotype than *shg^R64^* mutant embryos, with very big dorsal holes and lateral holes (Figure S2L). To further assess the role of DE-Cadherin on cell shape changes, we expressed different DE-Cadherin constructs in the epidermis, which have been shown to block cell shape changes in other contexts [13], [15], [69]. Nevertheless, none of these constructs phenocopied Rab11^DN^ in the epidermis.

In summary, Rab11^DN^ expression leads to DE-Cadherin mislocalization and lack of epidermal cell elongation, but our results do not support a direct relationship between these two phenotypes. We thus suggest that Rab11 is required for the growth of the apical membrane domain. We further propose that the lack of proper epidermal cell elongation increases the resistive force of the epidermis and this leads to detachments of the epidermis from the AS when adhesion is further compromised. This suggests that Rab11 is required in the epidermis to allow these cells to respond to the pulling force of AS cells.

#### Cell and tissue dynamics

To explore the effect of impaired Rab11 function on the dynamics of cell shape changes during DC we also performed time-lapse microscopy of ubi-DE-CadherinGFP embryos expressing Rab11^DN^ in the AS and/or the epidermis. In embryos expressing Rab11^DN^ in the AS only, cells showed straighter membranes than in wild type embryos (Figure 3C-0’ and Movie S6). These embryos complete DC but their kinetics of closure is different and zippering is impaired (K_z_ = 9.99 and 7.05 nm/sec). When expressed in the engrailed domain, Rab11^DN^ compromises the elongation of the epidermal cells (Figure 3F and Movie S7). In concert with this, the AS cells in contact with the engrailed stripes elongate as if they were being pulled back (Figure 3F-60’), which leads to the disruption of the tissue (Figure 3F-120’). This, together with the epidermal detachments observed in the *shg^R64^* mutants (Figure 3S), suggests that the epidermis requires Rab11 activity to elongate and respond to the pulling force of AS cell contraction.

In summary, our results show that Rab11-mediated recycling is required for proper AS and epidermal cell shape changes. These cannot be attributed solely to defects in DE-Cadherin localization, but are probably a consequence of perturbing membrane growth. Interestingly, Rab11 seems to be required in the epidermal cells to elongate in response to AS contraction.

### Endocytosis and recycling regulate AS apical membrane content, AS cell behaviour and tissue organization

**Figure 5:**
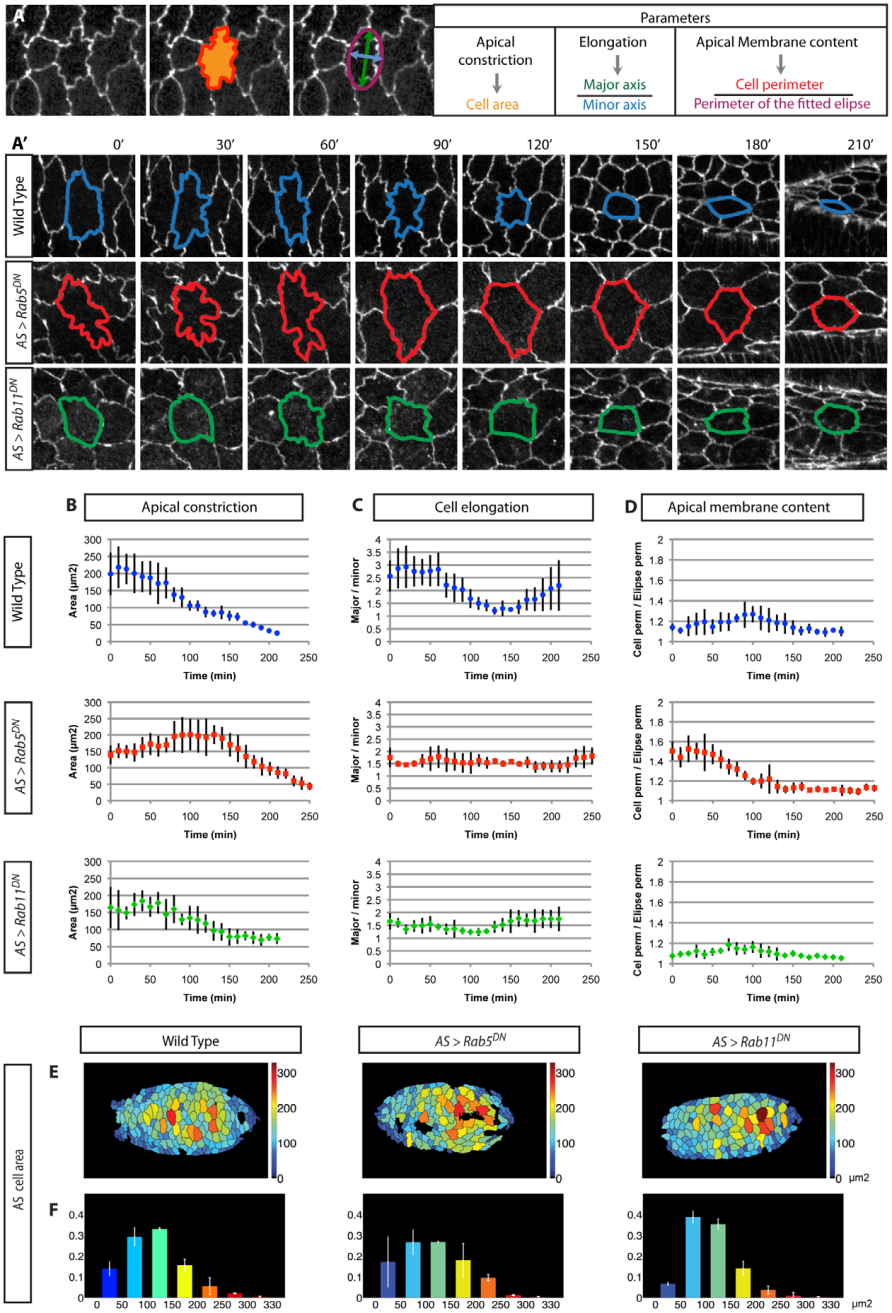
Quantification of AS apical membrane content, AS apical constriction and cell elongation. (A) Schematic representation of the three parameters measured in this study: apical surface area, elongation and membrane content. (A') Examples of AS cells followed in embryos of the indicated genotypes expressing Ubi-DE-CadherinGFP. (B–D) Quantification of apical surface area (B), cell anisotropy (C) and membrane content (D) for 5 cells of a representative embryo of each of the indicated genotypes. (E–F) Automated analyses of apical cell areas of the whole AS tissue in embryos at the beginning of DC. Cell area is shown with a colour code (E) and the proportion of cells in each cell area class is depicted in the bar graphs using the same colour code. In Rab5^DN^ expressing embryos, cells are more evenly distributed among the different cell area classes when compared with wild type (F). In Rab11^DN^ expressing embryos, the cell area is mostly concentrated in two cell area classes with small areas (F).

The AS has a central role in generating the force that drives DC. Interestingly, the above results from the qualitative analysis of AS cells after perturbing Rab5 and Rab11 function, suggest that endocytosis and recycling act in opposite ways on the morphology of AS cells during DC. To gain further insights into these differences we manually quantified the evolution of AS cell shape changes using three parameters: apical surface area, elongation and membrane content (Figure 5A and for details on analysis see Materials and Methods).

In wild type embryos, the average apical surface area of each AS cell exhibits a gentle decrease as DC progresses (Figure 5B). Cells with compromised Rab5 or Rab11 function exhibit much slower change of the same variable, though this is more pronounced in the case of Rab5 (Figure 5B). The fact that Rab11^DN^ expressing cells also show a slower apical constriction might reveal a need for AS cells to pass through the wiggly membrane state for correct cell behaviour and efficient apical constriction. Alternatively, Rab11 activity could be required to maintain correct levels of Cadherin molecules at the cell membrane which interacts with the cytoskeleton and drives efficient apical constriction.

We also measured the anisotropy of AS cells as the ratio between the major and minor axis of the best-fitted ellipse to each cell (Figure 5A). In wild type embryos AS cells are initially elongated in the D/V direction and then become more isotropic as they contract mainly in the D/V axis [60]. At later stages AS cells are more elongated in the A/P direction thus increasing again their anisotropy (Figure 5C). Such profile is not observed in embryos expressing Rab5^DN^ or Rab11^DN^ in the AS where the initial elongation is lower (average 1.5) than the one observed in wild type embryos (average between 2.5–3). AS cell elongation in these embryos is kept close to the initial values and does not exhibit a similar evolution to the one described above for wild type AS cells (Figure 5C). This analysis confirms that blocking Rab11 or Rab5 function in AS cells perturbs cell shape and has an effect on the overall organization of the tissue.

To complement our quantifications of apical cell area and gain some insight into the membrane content of the AS cells, we calculated the ratio between the real cell perimeter and the perimeter of the best-fitted ellipse (Figure 5A). In wild type embryos, AS cells are initially elongated in the D/V direction and exhibit straight membranes. As DC initiates, AS cell membranes become undulated giving rise to an increase in their apical membrane content. Towards later stages, this excess in the membrane content is reduced and cells exhibit again straighter membranes (Figure 5D). AS cells expressing Rab5^DN^ initially show a dramatic increase in their membrane content (approximately 50% more apical membrane than wild type AS cells), but as DC progresses these cells manage to reduce the excess of membrane (Figure 5D). In contrast, AS cells expressing Rab11^DN^ maintain a low membrane content throughout the process (Figure 5D). These results suggest that Rab5 and Rab11 modulate the amount of apical membrane during cell shape changes. In addition, it suggests that actomyosin-mediated apical constriction might be coupled to endocytosis to efficiently reduce the cell perimeter.

To complement the manual quantifications of a restricted number of AS cells over time, we also performed a semi-automated analysis of the entire AS tissue in embryos at the beginning of DC. This method provides more insight regarding the spatial cellular organization in the AS tissue and its constitution at this particular time point (Figures 5E and S5). Quantification of cell area revealed that in Rab5^DN^ expressing embryos there is a bigger variability in cell area, since cells are more evenly distributed among the different cell area classes when compared with wild type (Figure 5F). The cell area in Rab11^DN^ expressing embryos is mostly concentrated in two cell area classes, that correspond to small areas, suggesting that over the whole tissue cell size is more homogeneous (Figure 5F).

Altogether, the quantifications of the different parameters in the AS cells show that blocking Rab5 or Rab11 activity affects AS behaviour and tissue organization. In addition, it gives an estimation of the excess of apical membrane content in AS cells upon Rab5^DN^ expression and the decrease of membrane content in AS cells upon Rab11^DN^ expression.

### A balance between endocytosis and recycling is required to modulate cell behaviour during dorsal closure

**Figure 6:**
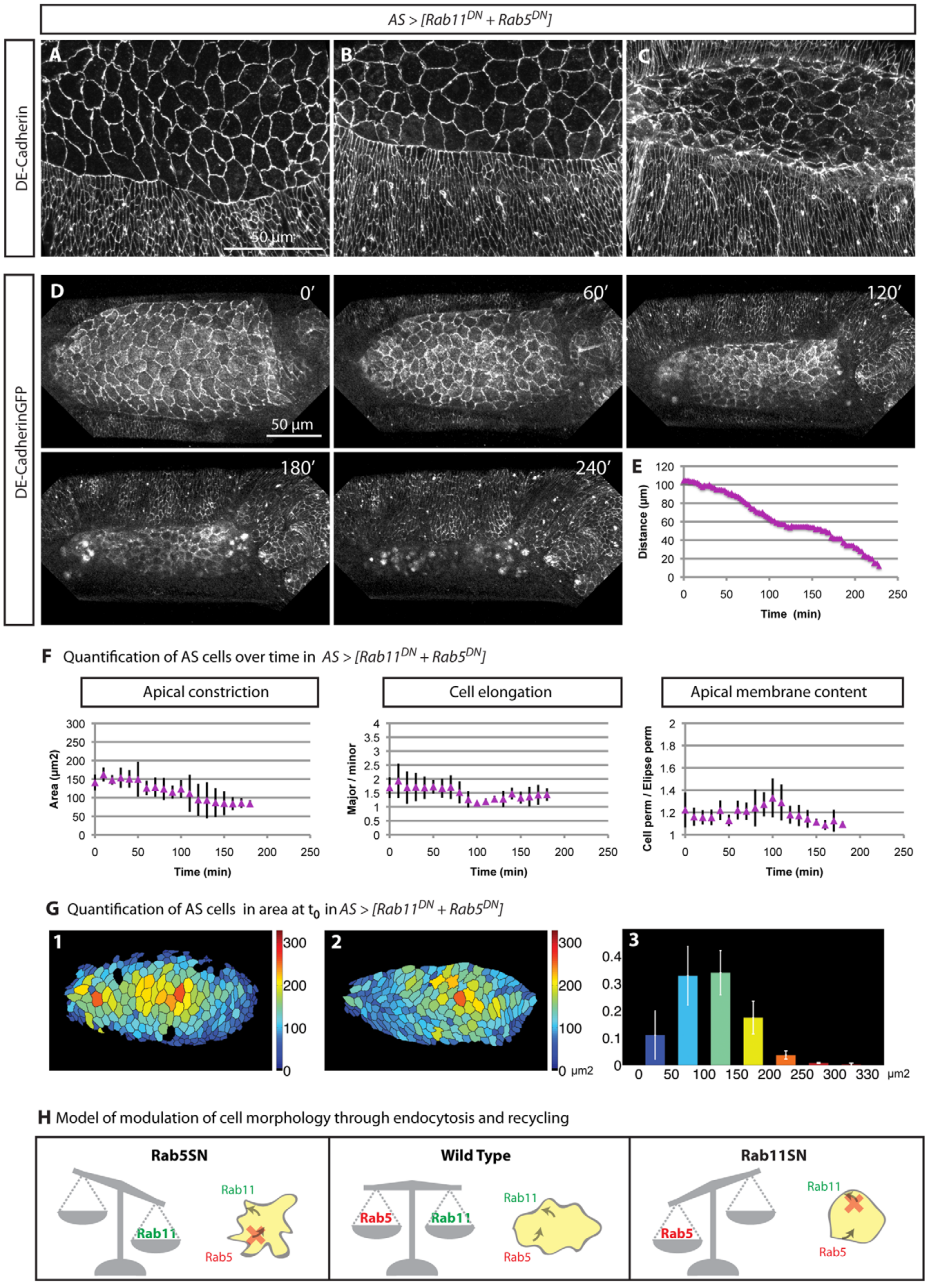
Balance between endocytosis and recycling modulates cellular morphology. (A-C) Confocal sections from *Drosophila* embryos expressing Rab5^DN^ and Rab11^DN^ simultaneously in the AS immunostained with an anti-DE-Cadherin antibody, showing cell shapes similar to the wild types. Still images from a time-lapse video of an embryo co-expressing Rab5^DN^ and Rab11^DN^ in the AS (D) and ubi-DE-CadherinGFP at indicated stages after the start of DC (Movie S8). (E) Measurement of the distance between the two opposing epithelia in these embryos. (F) Quantification of apical surface area, cell anisotropy and membrane content of 5 cells of 1 embryo over time. This embryo show features more similar to the wild type for the parameters analyzed, however zippering is defective and DC fails (D-240’). (G) Automated analysis of apical cell areas at the beginning of DC, as shown in Figure 5E–F. We found two different types of AS > [Rab11^DN^ + Rab5^DN^] embryos. In G1, an example embryo is depicted in which values for cell areas, direction of elongation and gradient of cell areas are close to wild type embryos. In G2 type embryos cells are smaller, the gradient is not as defined and cell elongation is random. The proportion of cells in each of the cell area classes is very similar to wild type (G3). (H) Model of the balance between endocytosis and recycling in cell morphology. When Rab5 mediated endocytosis is blocked there is less removal of membrane, which results in more membrane in AS cells and when Rab11 function is impaired, the insertion of membrane through Rab11 endosomes is blocked, and the apical membrane content is reduced.

The results presented above suggest that endocytosis and recycling are in an equilibrium that can be perturbed to modulate cell morphology (Figure 6H). A link between these two processes can be made on the account that endocytosis and recycling do not only target proteins but also membrane, the dynamics of which are key to AS behaviour. This proposal provides an explanation for the increase in apical membrane associated with blockage of Rab5 function resulting from a failure of endocytosis-mediated removal of membrane. Along the same lines, if Rab11 function is impaired, the insertion of membrane through Rab11 endosomes will be affected, and the apical membrane content is reduced, as we observed (Figure 6H). If our proposal is correct, we predict that blocking both processes simultaneously will result in wild type morphology, as the simultaneous interference of both processes will result in a balance. To test this, we expressed Rab5^DN^ and Rab11^DN^ simultaneously in the AS, and measured each of the three parameters that we have used to characterize these perturbations (Figure 6F). The results provide some support for our contention as indeed, the apical membrane content of the doubly perturbed cells approximates wild type. However, there must be other activities associated with Rab5 and Rab11 which do not balance each other as the majority of the embryos do not conclude DC (Figure S2A, I) and sometimes develop an abnormal organization of the AS (Figure 6A–D and Movie S8).

In fact, simultaneous expression of Rab5^DN^ and Rab11^DN^ in the AS led to different classes of phenotypes. This was specially clear with the semi-automated quantification of the whole AS (Figure 6 G1,2 and Figure S5), where some embryos exhibited deficient apical constriction and cell elongation (Figure 6F, G2). In contrast, in some embryos the tissue organization is indeed very similar to wild type, where a gradient of larger to smaller cells from the centre to the periphery of the tissue is observed and the proportion of cells in each of the cell area classes is very comparable to the wild type data (Figure 6 G1,3). This suggests once more that expressing both DNs simultaneously cancel their effects and results in almost wild type AS morphology. This observation supports the idea that Rab5 and Rab11 activities oppose each other and regulate cellular morphology and tissue organization. The fact that apical constriction is still affected when Rab5^DN^ or Rab11^DN^ are expressed simultaneously suggests that cells require a constant flux of membrane (endocytosis and recycling) which confers plasticity to the system and enables correct cell shape changes.

### Conclusions and future directions

Morphogenesis often involves interactions between tissues, which must coordinate and adjust their movements and properties to achieve their final shape. During DC, this can be seen in the anticorrelated behaviour of the epidermis and the AS: epidermal cells elongate and become flat increasing its surface area, while AS cells constrict apically and eventually disappear. This process is carefully choreographed under the influence of signalling pathways and mechanical constraints that impinge on the activity of the cytoskeleton and the adhesion system. While many studies have shown requirements for these systems, very little is known about the role of endocytosis and recycling in the AS and epidermal cell behaviour that characterises DC.

**Figure 7:**
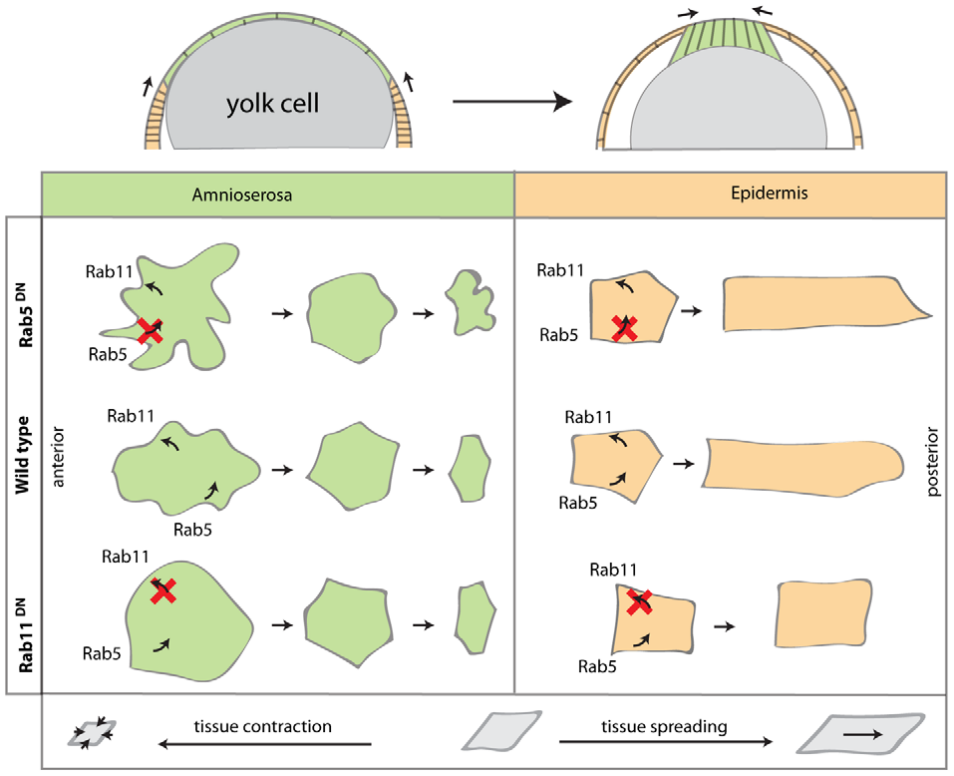
Different temporal requirements of endocytosis and recycling in the epidermis and AS. In wild type embryos AS cells are initially wiggly, then they reduce their apical surface area by contracting in the D/V direction, and this leads to reduction of AS tissue area. Epidermal cells elongate in the D/V direction which results in tissue spreading. If endocytosis is blocked, in the AS there is an increase in the apical membrane content and defects in apical surface reduction, indicating that Rab5 is required in the AS to remove membrane. In the epidermis it seems that expression of Rab5^DN^ does not have effects on epidermal cell shape changes. When recycling is blocked, AS cells exhibit straight boundaries at the onset of DC suggesting that Rab11 is required initially to maintain the membrane content of AS cells. In addition, Rab11 is required for epidermal cell elongation, which together with the observation in the AS cells, suggests that Rab11 is required for membrane growth.

Here we have used DN forms of Rab5 and Rab11 in mosaics to probe the specific contribution of these systems to the interactions between the AS and the epidermis during DC. We find that interfering with the traffic machinery has significant effects on the organization of the apical cell membrane in the two tissues. These effects are Rab- and tissue-specific (Figure 7). Our results suggest that the activity of Rab5 is required to remove membrane from the AS cells during apical constriction, whilst Rab11 exhibits an opposite effect, being required to increase the apical surface of cells. Based on the results obtained in DC we propose a model in which cells could control their shape by regulating the balance between endocytosis and recycling and we suggest this to be a mechanism widely used in morphogenesis to modulate cell shape changes (Figure 6H). However, in a complex morphogenetic process such as DC it is difficult to experimentally reproduce wild type cell shape changes when expressing Rab5^DN^ and Rab11^DN^ simultaneously. It would be thus interesting to test this model of the balance between endocytosis and recycling in cell culture where there are less inputs to account for.

This coupling between endocytosis and recycling [70], [71] raises the interesting possibility that the endocytic pathway might provide the molecular basis for a rheostat, which could be used to adjust the cortical activities and membrane dynamics of the AS and the epidermal cells. In this regard, from our results we suggest that in the AS, actin-myosin apical contraction could be coupled to Rab5-mediated endocytosis to remove apical membrane and effectively reduce apical cell area. Although it is not known how these two cellular processes interact, it will be interesting to measure the rate of membrane removal more directly and analyze how this process is coordinated with the fluctuating and contractile behaviour of AS cells.

We also suggest that the molecular machinery that promotes endocytosis and recycling can provide a system to mediate coordination between AS and epidermal tissue deformations. A revealing observation of our studies is the lack of epidermal cell elongation when Rab11 activity is blocked autonomously in the epidermis. A recent emphasis on the role of the AS in DC has led to a consideration of the epidermis as a passive passenger of the process that simply responds to the forces generated by the AS. Our observations indicate that the epidermal cells have to be capable of responding to the AS and that an element of this response is the ability of the epidermal cells to adjust their cortical and membrane systems through membrane trafficking.

Altogether, our results provide evidence that endocytic and recycling endosomes can act as membrane reservoirs required for cell shape changes during morphogenesis, (Lecuit and Pilot, 2003). They also suggest that the components of the traffic machinery could balance the activities of the cytoskeleton and the adhesion systems.

## Methods

### *Drosophila* strains

The following stocks were used in this study: wild type embryos were from the Oregon R strain, enGAL4 was used as an epidermal driver (although some AS cells could be seen expressing the corresponding reporter, indicating that this driver is leaky), c381GAL4 was used as a GAL4 driver expressed in the AS. As a ubiquitous driver, daGal4 was used. All of them were provided by the Bloomington stock center. UAS lines were expressed using the Gal4 system (Brand and Perrimon, 1993). Strains bearing the *shg^R64^* (Tepass et al., 1996) were provided by V. Hartenstein. We also used strains bearing the following transgenes: w;UASRab5SN/TM3 (Rab5^DN^), w;UASRab11SN/CyO (Rab11^DN^) and w;UASRab7TN/CyO (Rab7^DN^) provided by M. González-Gaitán, UASshiK44A/TM6B (Shi^DN^), provided by C. O’Kane, ubi-DE-CadherinGFP (Oda and Tsukita, 2001) and UASDE-CadherinGFP provided by F. Schweisguth. The following stocks were generated for this study: w;ubi-DE-CadherinGFP/CyO;UASRab5SN/TM6B, w;UASRab11SN,UASDE-CadherinGFP/CyO, w;UASRab11SN,shg^R64^/CyO, w;UASRab11SN,ubi-DE-CadherinGFP/CyO, w;UASRab11SN;UASRab5SN/SM6a-TM6B, w; UASRab11SN,UASDE-CadherinGFP;UASRab5SN/SM6a-TM6B.

### Immunostainings

Embryos were fixed and stained as previously described (Kaltschmidt et al., 2002). The following primary antibodies were used: rat anti-Ecad (DCAD2, 1/20, Developmental Studies Hybridoma Bank (DSHB)), rabbit anti-Rab5, Rab7 and Rab11 (1/25), kindly provided by M. González-Gaitán, rabbit anti-Rab5 (1/200, Abcam), mouse anti-Rab11 (1/100, BD Transduction Laboratories), mouse anti-Dynamin (1/100, BD Transduction Laboratories), mouse anti-β-tubulin (1/100, DSHB), rabbit anti-Zipper (MyosinII) (1/1000, R. Karess).

The following conjugated secondary antibodies were used: Alexa-fluor-488-, Alexa-fluor-568- and Alexa-fluor-647-conjugated antibodies from Molecular Probes and Alexa-fluor-568-conjugated phalloidin (Molecular Probes) for F-actin detection.

Fluorescently labelled embryos were mounted in Vectashield (Vector) and examined under a Nikon D-Eclipse C1 confocal scanning unit, mounted on a Nikon Eclipse 90i microscope, using the EZ-C1 3.60 software and a 60x/1.40 NA Apo VC immersion-oil objective.

### Quantification of Rab protein, cell elongation and DE-Cadherin levels

Fluorescence intensity levels of Rab5 and Rab11 in Figure 1 were quantified in AS and epidermal cells after elongation using the plot profile function from ImageJ (http://rsb.info.nih.gov/ij/). An area of 60×40 µm was quantified on the original images from Figure 1B″, E″. Cell elongation of epidermal cells was measured in the DME cells using ImageJ. Wild type cells and mutant cells from embryos expressing Rab11^DN^ or Rab11^DN^ + UASDE-CadherinGFP were compared using One-Way ANOVA and Tukey HSD Test for Post-ANOVA Pair-Wise Comparisons. DE-Cadherin fluorescence intensity values were transformed into specific colours along the gradient shown in Figure 4 using LUT Fire in ImageJ.

### Cuticle preparations

Embryos were collected from 24-hour-old eggs and then aged 24 hours at 25°C. They were dechorionated in bleach, mounted in acetic acid:Hoyers (1∶1) and the slide was incubated overnight at 65°C.

### Time-lapse movies

Stage 13 *Drosophila* embryos carrying an ubi-DE-CadherinGFP construct (Oda and Tsukita, 2001), were dechorionated, mounted on coverslips with the dorsal side glued to the glass and covered with Voltalef oil 10S (Attachem). Imaging of the embryos was done using an inverted LSM 510 Meta laser-scanning microscope with a 40X oil immersion Plan/Fluor (NA = 1.3) objective or 63X oil immersion Plan-Apochromat (NA = 1.4). Embryos were maintained at 24°C during imaging and around 50 *z*-sections 1 µm apart were collected every 2 minutes.

### Quantification of time-lapse movies

Manual quantification of 5 cells per embryo of each genotype (wild type, embryos expressing Rab5^DN^, Rab11^DN^ or both simultaneously in the AS) was performed using ImageJ. Every 10 min in the time-lapse the boundaries of the cells were drawn and an ellipse that has the same area as the cell and an approximated geometry was obtained using the command Fit ellipse. The area, perimeter of the cell, major and minor axis were obtained with the Measure command and different parameters were calculated as described in Figure 5A.

In the semi-automated quantification we determined the distributions of the geometrical properties of the AS at the onset of DC of the different genotypes. The live images were segmented applying the watershed algorithm in Matlab, which in some cases created oversegmentation that we corrected manually. From the segmented image we were able to calculate the apical cell areas.

### Dextran uptake assays

Embryos at DC stage were selected and aligned with the ventral region upward and anterior part towards the observer on top of a narrow stripe of double-sided tape. Sörensen phosphate buffer (SPB) was added to cover the aligned embryos. The vitelline membrane was pierced at the head with a glass needle that was subsequently moved to the posterior of the embryo, and teased out of the vitelline membrane, away from the tape. These hand-devitellinized embryos were transferred with a coated glass pipette into a coated glass dish with 3.7 mM of Dextran Texas Red, 3 000 MW, lysine fixable from Invitrogen, diluted in SPB and incubated for 1 hour at 4°C. Then, they were rinsed and washed several times with SPB at 4°C and chased for 15 minutes in SPB at 25°C. Fixation was performed in paraformaldehyde (PFA) 4% for 40 minutes at 25°C and then wash-blocked in BBT-BSA (BBS + CaCl2 1 mM + 0,1% Triton + 0,5% BSA). For further antibody labelling, embryos were incubated with other primary antibodies diluted in BBT-BSA for 2 hours at RT, and thoroughly washed with BBT-BSA. Finally, embryos were incubated with BBT-BSA containing secondary antibodies at RT for 2 hours in the dark, rinsed and washed several times in BBT-BSA and then individually mounted in Vectashield.

## Supporting Information

Figure S1 **Blocking Rab11 simultaneously in the AS and epidermis leads to the expansion of AS cells.** Time lapse of embryos expressing DE-CadherinGFP ubiquitously shows the AS apical reduction and epidermal elongation during DC (A). In embryos expressing Rab11^DN^ ubiquitously, epidermal cells fail to elongate, AS cells expand over time and less DE-Cadherin is detected at the apical membrane (B'). Eventually, holes are formed between AS cells (B-180’, 210’, arrowhead) and AS detaches from the epidermis (B-240’ arrow).
(TIF)

Figure S2 **Cuticles laid by embryos expressing the different DNs.** (A) Quantification of embryonic lethality and cuticle phenotypes of embryos expressing Rab5^DN^, Rab11^DN^, Rab7^DN^, Shi^DN^ or Rab5^DN^+ Rab11^DN^ ubiquitously, in the AS or in epidermal stripes. (B-J) Embryonic cuticles of the predominant phenotypes. Arrows indicate the grooves in the cuticles. NA- Not Analysed.
(TIF)

Figure S3 **Dextran uptake assays in Epidermal>Rab5^DN^ embryos and Rab5-independent processes for membrane removal.** Expression of Rab5^DN^ in *Drosophila* embryos blocks endocytosis (A-C). Embryos expressing Rab5^DN^ in the engrailed domain were hand-devitellinized and incubated in Dextran for 1 hour at 4°C, chased for 15 minutes at RT, then fixed and stained for Rab5. Expression of Rab5^DN^ leads to a more diffused labelling of the Rab5 protein. Cells expressing Rab5^DN^ (in green) exhibit reduced uptake of Dextran (B) when compared with cells not expressing the DN. AS cells show fine projections in embryos expressing Ubi-DE-CadherinGFP and Ubi-DE-CadherinGFP + AS > Rab5^DN^ (D,E). Folding of AS cell membrane is also observed in embryos of both genotypes (F-I).
(TIF)

Figure S4 **The effects of Dyn^DN^ are similar to Rab11^DN^ but do not phenocopy Rab5^DN^.** Blocking Dynamin ubiquitously disrupts epithelia integrity similarly to Rab11^DN^ (A-E). Blocking Dynamin in the AS has different effects from blocking Rab5 (F-H'). Apical constriction in AS cells appears affected (G, H), but there is no increase in the membrane undulations at early stages (F) nor very big lamelipodia (F'-H').
(TIF)

Figure S5 **Automated analyses of the whole AS tissue in embryos at the beginning of DC.** Remaining embryos used in the bar graphs with the proportion of cells of each lass of cell area (Figure 5 and 6). Cell area is shown with a colour code.
(TIFF)

Movie S1 Time-lapse of Ubi-DE-CadherinGFP embryos.
(AVI)

Movie S2 Time-lapse of Ubi-DE-CadherinGFP + AS > Rab5^DN^ embryos.
(AVI)

Movie S3 Time-lapse of Ubi-DE-CadherinGFP + AS > Rab5^DN^ embryos.
(AVI)

Movie S4 Time-lapse of Ubi-DE-CadherinGFP embryos.
(AVI)

Movie S5 Time-lapse of Ubi-DE-CadherinGFP + Epidermal > Rab5^DN^ embryos.
(AVI)

Movie S6 Time-lapse of Ubi-DE-CadherinGFP + AS > Rab11^DN^ embryos.
(AVI)

Movie S7 Time-lapse of Ubi-DE-CadherinGFP + Epidermal > Rab11^DN^ embryos.
(AVI)

Movie S8 Time-lapse of Ubi-DE-CadherinGFP + AS > [Rab11^DN^ + Rab5^DN^] embryos.
(AVI)
