# Supplementary figures and images for: Endocytic and Recycling Endosomes Modulate Cell Shape Changes and Tissue Behaviour during Morphogenesis in Drosophila
Source: PLoS One. 2011 Apr 14;6(4):e18729. doi: 10.1371/journal.pone.0018729 (PMC3077405; doi:10.1371/journal.pone.0018729)

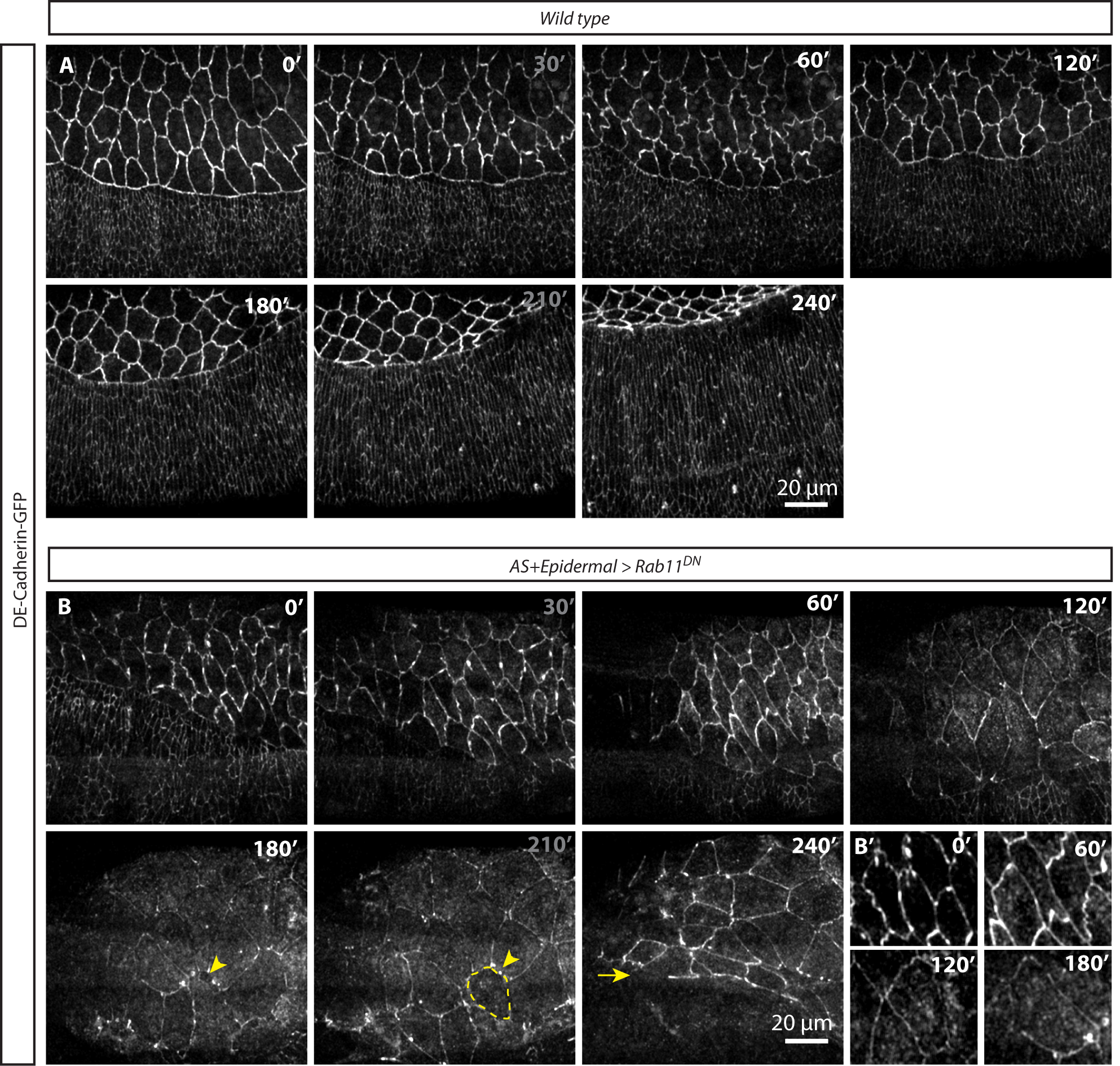

Supplement: Figure S1 — Blocking Rab11 simultaneously in the AS and epidermis leads to the expansion of AS cells. Time lapse of embryos expressing DE-CadherinGFP ubiquitously shows the AS apical reduction and epidermal elongation during DC (A). In embryos expressing Rab11DN ubiquitously, epidermal cells fail to elongate, AS cells expand over time and less DE-Cadherin is detected at the apical membrane (B'). Eventually, holes are formed between AS cells (B-180’, 210’, arrowhead) and AS detaches from the epidermis (B-240’ arrow). (TIF) [file pone.0018729.s001.tif]

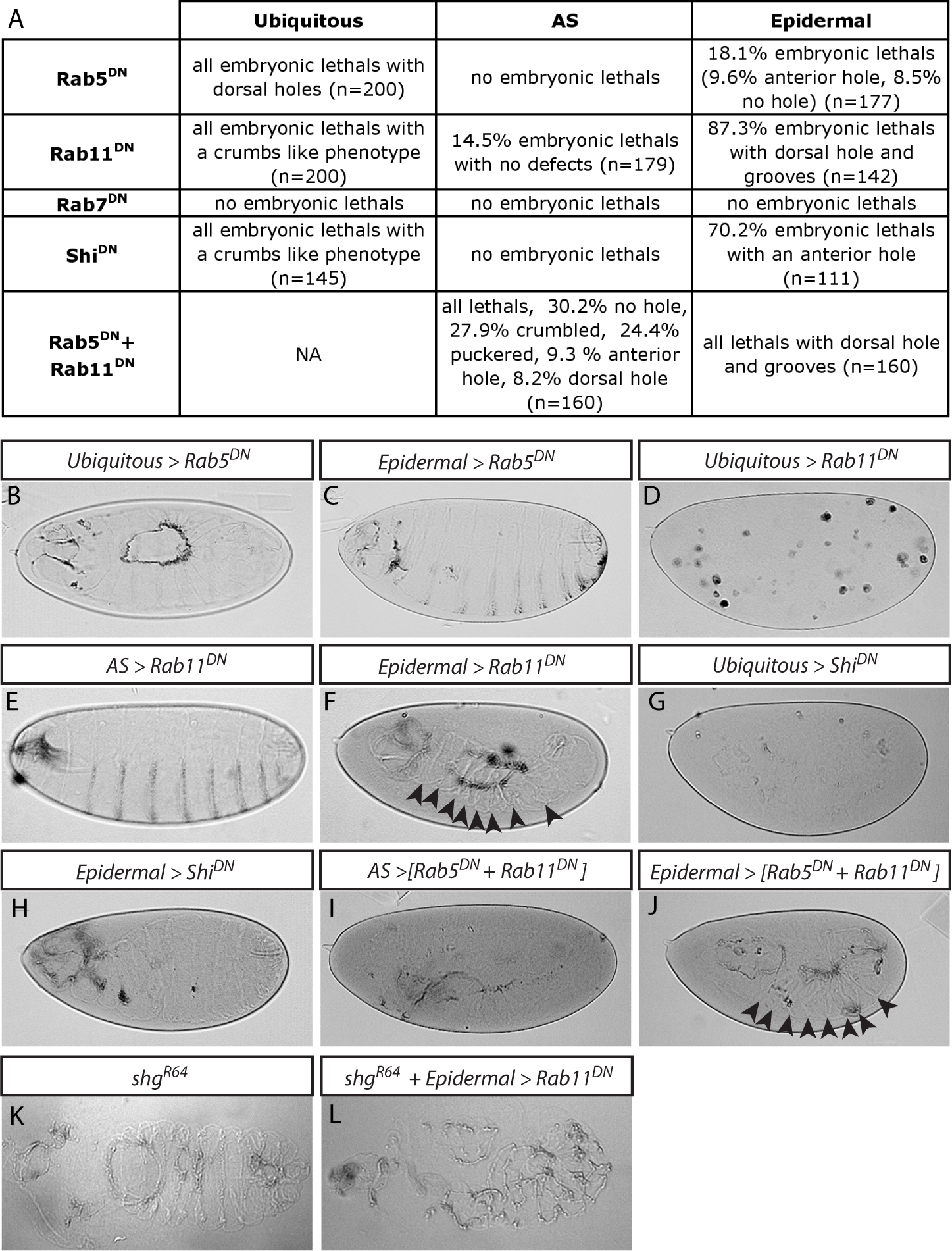

Supplement: Figure S2 — Cuticles laid by embryos expressing the different DNs. (A) Quantification of embryonic lethality and cuticle phenotypes of embryos expressing Rab5DN, Rab11DN, Rab7DN, ShiDN or Rab5DN+ Rab11DN ubiquitously, in the AS or in epidermal stripes. (B-J) Embryonic cuticles of the predominant phenotypes. Arrows indicate the grooves in the cuticles. NA- Not Analysed. (TIF) [file pone.0018729.s002.tif]

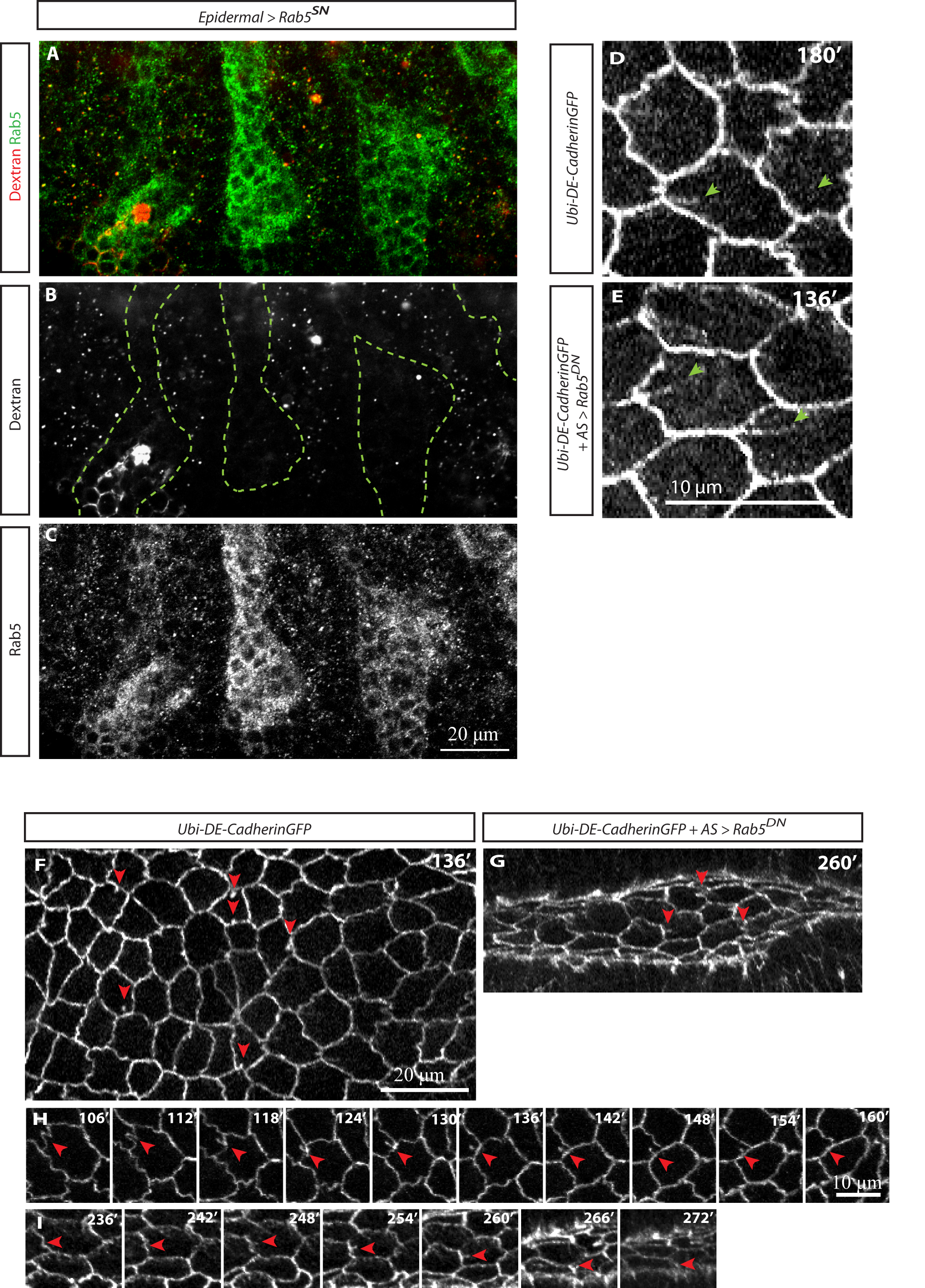

Supplement: Figure S3 — Dextran uptake assays in Epidermal>Rab5DN embryos and Rab5-independent processes for membrane removal. Expression of Rab5DN in Drosophila embryos blocks endocytosis (A-C). Embryos expressing Rab5DN in the engrailed domain were hand-devitellinized and incubated in Dextran for 1 hour at 4°C, chased for 15 minutes at RT, then fixed and stained for Rab5. Expression of Rab5DN leads to a more diffused labelling of the Rab5 protein. Cells expressing Rab5DN (in green) exhibit reduced uptake of Dextran (B) when compared with cells not expressing the DN. AS cells show fine projections in embryos expressing Ubi-DE-CadherinGFP and Ubi-DE-CadherinGFP + AS > Rab5DN (D,E). Folding of AS cell membrane is also observed in embryos of both genotypes (F-I). (TIF) [file pone.0018729.s003.tif]

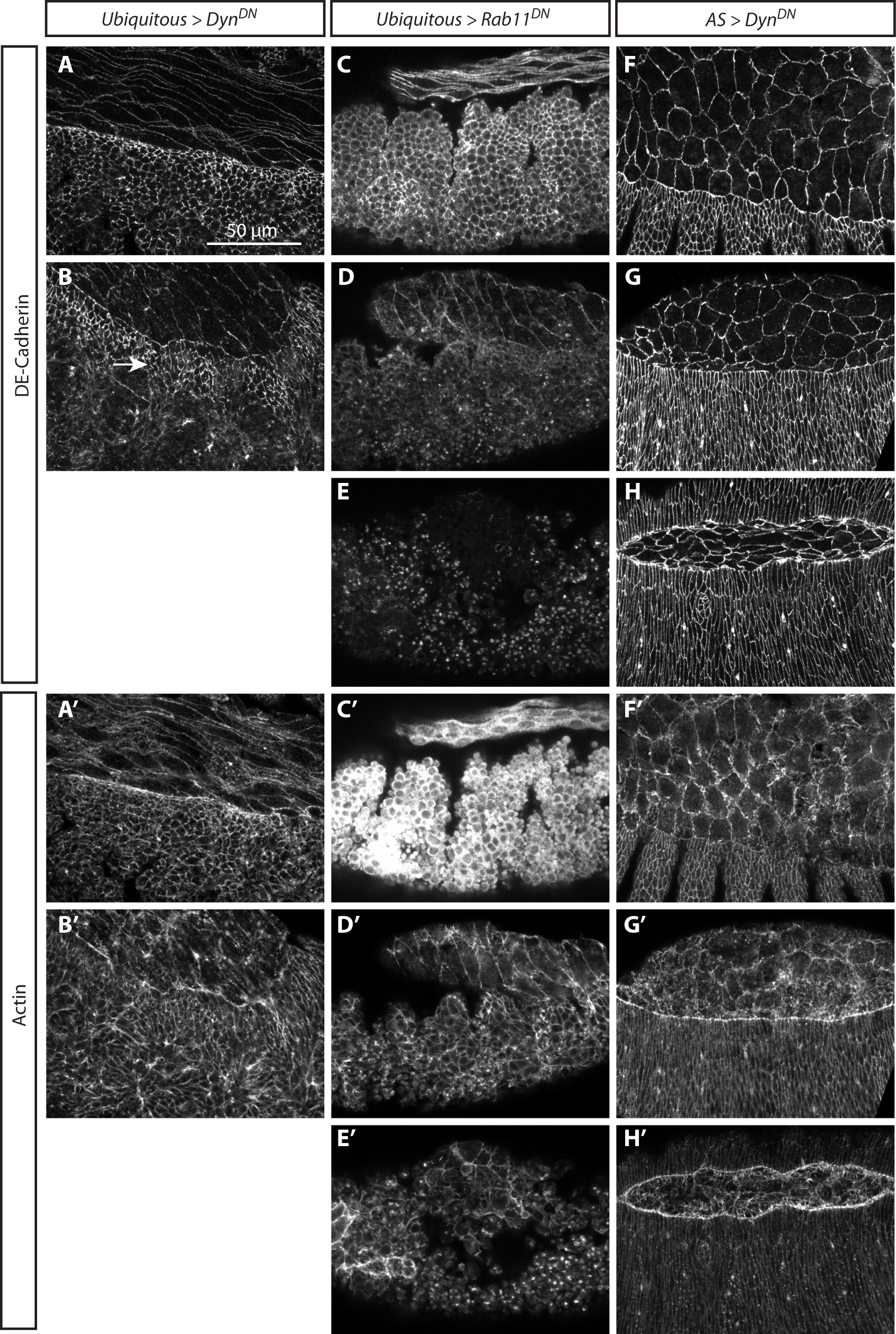

Supplement: Figure S4 — The effects of DynDN are similar to Rab11DN but do not phenocopy Rab5DN. Blocking Dynamin ubiquitously disrupts epithelia integrity similarly to Rab11DN (A-E). Blocking Dynamin in the AS has different effects from blocking Rab5 (F-H'). Apical constriction in AS cells appears affected (G, H), but there is no increase in the membrane undulations at early stages (F) nor very big lamelipodia (F'-H'). (TIF) [file pone.0018729.s004.tif]

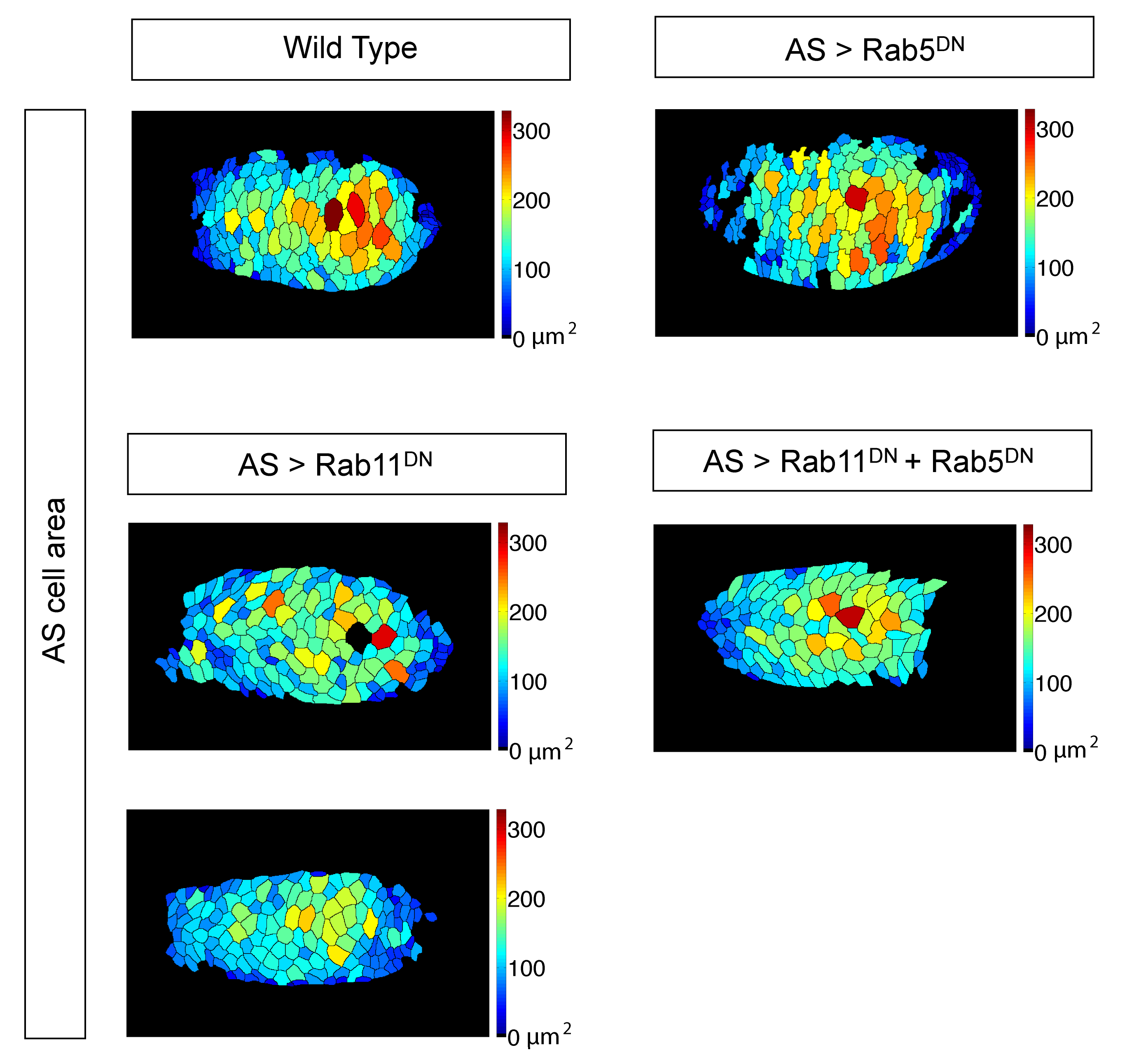

Supplement: Figure S5 — Automated analyses of the whole AS tissue in embryos at the beginning of DC. Remaining embryos used in the bar graphs with the proportion of cells of each lass of cell area (Figure 5 and 6). Cell area is shown with a colour code. (TIFF) [file pone.0018729.s005.tif]
